# Estimating the transfer function of cortical neurons : from simple models to in vitro experiments

**DOI:** 10.1186/1471-2202-14-S1-P105

**Published:** 2013-07-08

**Authors:** Yann Zerlaut, Charlotte Deleuze, Gilles Ouanounou, Thierry Bal, Alain Destexhe

**Affiliations:** 1 Unité de Neurosciences, Information and Complexité, CNRS, Gif-sur-Yvette 91198, France

## 

Important features of neural network activity can be described with mean-field formalisms. The core of those descriptions are the neuronal transfer functions, i.e. a function that maps the output firing rate of a neuron to a given synaptic input. To design realistic mean-field models, one must estimate the transfer function from real neurons, but unfortunately this is not possible because scanning the synaptic input space would require to keep a stable recording for many hours. One way to solve this problem is to obtain an analytic template that can fit the transfer function of complex neuron models, and use this template to guide the experiments. This is the approach that we have followed in this study. We found that the same analytic template derived from current-based models can also fit well the transfer function of more complex models, if the parameters are rescaled appropriately. The procedure was succesfull for model of increasing complexity from conductance-based Integrate-and-Fire to Hodgkin-Huxley models with adaptation (models of regular-spiking cells), which suggests that it may also work for real neurons. Based on this approach, we have performed experiments to calculate the transfer function from real neurons in rat cortical slices. We used the dynamic-clamp setup to inject different combinations of excitatory and inhibitory conductance inputs. With a limited number of sampling points, we were able to determine the transfer function of V1 neurons. This approach opens the way towards building realistic mean-field models of cortical networks.

